# Sleep disturbances in diabetic peripheral neuropathy patients: a clinical and polysomnographic study

**DOI:** 10.1186/s41983-018-0024-0

**Published:** 2018-09-04

**Authors:** Wafik Said Bahnasy, Yasser Abo Elfotoh El-Heneedy, Ehab Ahmed Shawky El-Seidy, Nema Ata Allah Labib, Ibrahim Salah Eldeen Ibrahim

**Affiliations:** 1 Department of Neuropsychiatry, Tanta University, Tanta, 31527 Egypt; 2 Department of Chest Diseases, Tanta University, Tanta, 31527 Egypt; 3 Department of Neuropsychiatry, Faculty of Medicine, Tanta University, Tanta, 31511 Egypt

**Keywords:** Diabetic peripheral neuropathy, Diabetic autonomic neuropathy, Sleep apnea syndrome, Multiple sleep latency test and polysomnography

## Abstract

**Background:**

Disordered sleep breathing is a common complication of diabetic peripheral neuropathy (DPN) manifested by excessive daytime sleepiness, morning headache, morning dizziness, cognitive decline, and mood changes.

**Methods:**

This study was performed on 30 non-obese type 2 diabetic patients; 20 with clinically evident DPN and 10 without. Ten age-, sex-, and body mass index-matched healthy control subjects were also included. Patients and control were subjected to history taking, neurological examination, glycated hemoglobin, and clinical assessment of the sensori-motor manifestations by the neuropathy symptom score and neuropathy disability score. The autonomic nervous system was evaluated clinically by the systolic blood pressure response to standing and heart rate response to each of standing, Valsalva, and deep breath. Finally, sleep was assessed by one-night polysomnogram (PSG) followed by multiple sleep latency test in the next day.

**Results:**

The study showed significant increase in sleep apnea syndromes in diabetic peripheral neuropathy patients compared to diabetic neuropathy free patients and healthy control (*p* < 0.0001). The sleep apnea was mainly obstructive and to a little extent mixed (obstructive/central) sleep apnea. The severity of sleep PSG abnormalities was positively correlated with the severities of sensory, motor, and autonomic manifestations.

**Conclusions:**

Non-obese type 2 diabetic patients complicated by peripheral neuropathy especially those having dysautonomia are at increased risk of developing sleep disordered breathing resulting in their excessive daytime sleepiness, decreased productivity, and poor glycemic control.

## Background

Diabetic peripheral neuropathy (DPN) is one of the most common complications of diabetes (DM) affecting 20–60% of type 2 diabetic patients (Qu et al. 2017). It is defined as somatic and/or autonomic neuropathy attributed solely to DM. Diabetic autonomic neuropathy (DAN) is the most under-diagnosed, yet one of the most serious complications of DM caused by damage of efferent parasympathetic and/or sympathetic nerves (Feldman et al. 2017). Diabetic autonomic neuropathy is usually of insidious onset in long standing poorly controlled diabetics (Villafaina et al. 2017). Its most common manifestations include resting tachycardia, postural hypotension, arrhythmias, gastroparesis, diarrhea, constipation, gustatory sweating, dry skin, erectile dysfunction, retrograde ejaculation, atonic bladder, abnormal renal sodium handling, pupillary dysfunction, and bronchoconstriction (Martin et al. 2014).

There is a reciprocal relationship between sleep apnea syndromes (SAS) and DM while the presence of DAN increases this negative interaction. Patients with DAN have nocturnal symptoms interfering with their sleep quality and are at higher risks of sudden death during sleep up to fivefolds than those without (Pop-Busui et al. 2010). On the other hand, SAS in diabetics result in increased insulin resistance, impaired glucose tolerance, and excessive daytime sleepiness (EDS) which results in patients’ easy fatigue, cognitive decline, mood changes, and reduced productivity (Schober et al. 2011).

### Aim of the work

Was to study the possible existence of sleep abnormalities in DPN patients and their relations to the severities of both sensori-motor and autonomic manifestations.

## Methods

The present study was conducted on 30 non-obese type 2 diabetic patients attending the neurology and internal medicine diabetes clinics, Tanta University Hospitals, in the period between 1st of April and 1st of December 2015. Patients were divided into two groups; group I included 20 clinically evident sensori-motor DPN patients with or without autonomic manifestations and group II included 10 diabetic neuropathies free (DNF) patients. The study also included 10 healthy control subjects (group III) matching the patients’ age, sex, and body mass index (BMI).

Exclusion criteria invloved patients with respiratory or cardiac problems, chronic pain, and advanced metabolic, neuropsychiatric or endocrinal disorders affecting sleep and patients with BMI > 28 and history of medications intake affecting sleep and who are heavy smokers or drug abusers.

The protocol of the study was approved by The Research Ethics Committee and Quality Assurance Unit, Faculty of Medicine, Tanta University. Participation was voluntary, all participants received detailed information concerning the aims of the study and the possible risks, and an informed consent was obtained from all prior to the commencement in the study.

Diabetes mellitus was diagnosed according to American Diabetes Association 2015 (Polonsky and Burant 2016). Patients and controls were submitted to history taking, neurological examination, and routine laboratory investigations. Clinical diagnosis of DPN and grading of its severity was done using the neuropathy symptom score (NSS) and neuropathy disability score (NDS). A total NSS of 3–4 considered mild, 5–6 moderate, and 7–9 severe symptoms. A total NDS of 3–5 considered mild, 6–8 moderate, and 9–10 severe disability. Neuropathy was diagnosed in patients with moderate disability with or without symptoms, or mild disability with moderate symptoms. Mild disability and/or mild symptoms were not considered adequate to diagnose clinical evident DPN (Kisozi et al. 2017; Cabezas-Cerrato 1998). Dysautonomia was clinically assessed by measuring systolic blood pressure response to standing and heart rate response to each of standing, Valsalva, and deep breath.

All subjects were submitted to one-night polysomnogram (PSG) followed in the next day by multiple sleep latency test (MSLT) which is an objective assessment of EDS (Littner et al. 2005). PSG was performed by a Somon Medics Gmbh (Am SonnenstuhL63, D-97236 Rander Sacker, Germany, Type: SOMNO screen™plus, SN: 4259, kw45: 2014). Each PSG included EEG channels montages (O1/A2, C3/A2, C4/A1 and O2/A1), electrooculography (LOC-A1/A2 and ROC-A1/A2), surface tibial and submental EMG, and modified V2 lead ECG. For respiratory sensors, nasal and oral signals by thermal airflow sensors (thermistor) were used, tracheal sounds microphone was applied, and the chest and abdominal effort was measured by dual thoracoabdominal RIP (respiratory inductance plethysmography) belts.

The studied parameters were scored according to The American Academy of Sleep Medicine Scoring Manual, 2012 (Grigg-Damberger 2012). Sleep latency (SL) refers to the length of time taken in transition from wakefulness to sleep, wake after sleep onset (WASO) is the minutes of wake after sleep onset but before the final awakening, sleep efficiency (SE) is the total sleep time (TST) divided by the total in bedtime, sleep fragmentation (SF) is the number of sleep cycles/night, and sleep stage transition index (SSTI) is the number of transition between various sleep stages/hour. The pulse transit time (PTT) is the time taken for the arterial pulse wave to travel from the aortic valve to a peripheral site which reflects the intrathoracic pressure. An arousal refers to abrupt shift of EEG activities which last for ≥ 3 s and are preceded by ≥ 10 s of sleep. An apnea was defined as ≥ 90% drop in the thermistor excursion signal of ≥ 10 s and hypopnea which was defined as a decrease in thermistor signal by > 30% for > 10 s accompanied by a decrease of oxyhemoglobin saturation ≥ 4%. The patient was considered to have sleep apnea syndrome (SAS) if the apnea hypopnea index (AHI) is ≥ 15 or if the AHI ≥ 5 associated with insomnia, cardiovascular comorbidities, or diurnal symptoms mainly EDS, impaired cognition, or mood changes (Sateia 2014).

Statistical analysis was conducted using SPSS version 19 (Statistical Package for Social Studies) created by IBM, Chicago, IL, USA. For numerical values, the range and mean ± standard deviations were calculated. For categorical variable, the number and percentage were calculated and differences between subcategories were tested using the *z*-score test, ANOVA, Tukey’s tests, and post-hoc tests. Correlation analysis was performed using Pearson’s correlation test. *p* value < 0.05 was considered statistically significant.

## Results

The study included 20 DPN patients, with mean ages 48.25 ± 7.7 years, mean BMI 25.7 ± 1.97 kg/m^2^, 12 females (60%) and 8 males (40%). Groups II and III were selected to be age, sex and BMI matched with DPN group. Diabetes duration (years) and glycated hemoglobin % were significantly higher in DPN group than DNF group with *p* value = 0.0003 and < 0.0001 respectively (Table 1). Diabetes treatment regimens in DPN patients were changeable and cannot be correlated with sleep changes, and at the time of research, 10 patients were on different types of oral hypoglycemic drugs, 4 on insulin, and 6 on combination therapy.

**Table 1:** Glycated hemoglobin, neuropathy symptom and disability scores, and autonomic nervous system assessment among diabetic neuropathy patients (group I), diabetic patients without neuropathy (group II) and healthy control (group III)

	Group I		Group II		Group III		ANOVA	
	Range	Mean ± SD	Range	Mean ± SD	Range	Mean ± SD	*f* value	*p* value
HBA1c (%)	6.7–11.5	8.33 ± 1.48	6.8–8.5	7.57 ± 0.53	5.5–6.3	5.90 ± 0.26	16.18	< 0.0001*
NSS	5–9	7.1 ± 1.48	1–4	2 ± 1.05	1–3	1.6 ± 0.69	92.73	< 0.0001*
NDS	6–10	8.05 ± 1.5	1–4	2.1 ± 0.99	1–2	1.5 ± 0.52	133.6	< 0.0001*
PSH	10–40	23.3 ± 11.2	5–13	8.8 ± 2.48	5–10	6.7 ± 1.49	18.46	< 0.0001*
Postural tachycardia	13–39	23.8 ± 8.03	13–26	14.3 ± 4.5	11–21	15.3 ± 2.98	6.31	0.0044*
HRDB	5–16	10.7 ± 3.4	9–18	13.1 ± 2.76	9–18	13.9 ± 2.88	4.08	0.025*
HRV	1.1–1.3	1.25 ± 0.06	1.3–1.4	1.29 ± 0.04	1.2–1.4	1.29 ± 0.04	3.40	0.51

*HRDB* heart rate response to deep breath, *HRV* heart rate response to Valsalva, *NDS* neuropathy disability score, *NSS* neuropathy symptom score, *PSH* postural systolic hypotension

*Significant

History and examinations of DPN group revealed 9 patients (45%) had slippering of slippers, 8 patients (40%) had postural dizziness, and 11 patients (55%) had urinary symptoms. Five male patients (25%) had erectile dysfunctions, 1 patient (5%) had history of diabetic ophthalmoplegia, 4 patients (20%) had gustatory sweating, 5 patients (25%) had peripheral limb ischemia, and 7 patients (35%) had gastrointestinal symptoms mainly bloating, gastric distension, and constipation alternating with diarrhea.

Neuropathy symptom score among DPN group was 7.1 ± 1.48 compared to 2 ± 1.05 and 1.6 ± 0.69 in DNF and control groups respectively with *p* < 0.0001. Moreover, NDS in DPN group were 8.05 ± 1.5 compared to 2.1 ± 0.99 and1.5 ± 0.52 in DNF and control groups respectively with *p* < 0.0001 (Table 1).

Thirteen DPN patients (65%) had objective autonomic manifestation in one of the included examinations. The study showed that, postural systolic hypotension, postural tachycardia, abnormal heart rate response to deep breath were significantly higher in DPN group compared to DNF and control groups. Heart rate response to Valsalva showed non-significant difference between the three studied groups (Table 1). The degree of dysautonomia was directly proportional with disease duration, HBA1c %, and severity of sensori-motor manifestations measured by each of NSS and NDS.

Regarding sleep architecture, 8 DPN patients (40%) had SAS which was significantly higher than each of DNF and control groups (10% in each). In the DPN group, 6 patients had obstructive sleep apnea (OSA) and 2 had mixed sleep apnea (MSA) while the SAS subjects in groups 2 and 3 were OSA only. In a parallel way, the AHI was significantly higher in group I than groups 2 and 3 with *p* value < 0.0001 (Table 2).

**Table 2:** Sleep parameters among diabetic neuropathy patients (group I), diabetic patients without neuropathy (group II) and healthy control (group III)

	Group I		Group II		Group III		ANOVA	
	Range	Mean ± SD	Range	Mean ± SD	Range	Mean ± SD	*f* value	*p* value
Mean latency MSLT (min)	4–17	9.2 ± 4.03	10–18	15.1 ± 3.44	12–19	16.30 ± 2.21	17.27	< 0.0001*
Sleep period time (hours)	5.2–8.7	6.79 ± 1.06	5.3–8.6	6.79 ± 1.03	5.2–8.7	6.78 ± 1.09	0.00066	0.999
Total sleep time (hours)	4.8–8.5	6.29 ± 1.07	5. –8.4	6.70 ± 1.04	5.2–8.5	6.68 ± 1.05	0.692	0.5068
Sleep latency (min)	4–7	8.45 ± 4.04	7–35	24.70 ± 9.38	8–35	26.3 ± 8.82	29.96	< 0.0001*
Sleep efficiency (%)	49–85	63.3 ± 11.78	73–88	80.6 ± 4.27	77–94	85.8 ± 6.14	24.11	< 0.0001*
Sleep fragmentation (cycles/night)	7–43	18.35 ± 9.06	6–15	9.4 ± 2.71	6–11	8.2 ± 1.54	10.30	0.0003*
Apnea Hypopnea Index (/hour)	3.1–83.3	31.32 ± 16.4	1.2–22.1	6.57 ± 7.01	1.6–19.2	5.97 ± 6.14	16.199	< 0.0001*
Pulse Transit Time (msec)	121–245	184 ± 23.2	154–276	232 ± 12.3	178–281	241 ± 13.2	13.6	< 0.0001*
Desaturation index (/hour)	3–32	14.9 ± 9.7	2–8	3.8 ± 1.87	2–7	3.7 ± 1.6	12.55	< 0.0001*
Lowest O_2_ saturation (%)	71–95	85.15 ± 3.78	87–94	90.9 ± 2.55	87–95	92.5 ± 2.50	6.522	0.0023*
REM latency (min)	64–105	84.7 ± 12.38	63–102	85.4 ± 11.82	65–104	85.70 ± 12	0.0259	0.974
REM % of TST	8–21	14 ± 4.30	18–25	21.8 ± 2.04	18–27	21.1 ± 2.02	24.10	< 0.0001*
Arousal index (/hour)	6–35	13.8 ± 7.39	3–14	6.5 ± 3.02	3–11	5.5 ± 2.63	9.585	0.0004*
Snore index (/hour)	23–632	259.3 ± 174	10–67	29.9 ± 19.8	9–47	17 ± 11.2	17.76	< 0.0001*
Snore % TST	4–52	23.4 ± 14.27	3–13	6.1 ± 3.07	2–10	4.6 ± 3.27	14.93	< 0.0001*
PLMs index (/hour)	17–91	50.3 ± 22.6	5–27	14.4 ± 6.73	4–18	9.9 ± 6.66	27.60	< 0.0001*

*MSLT* multiple sleep latency test, *PLMs* periodic limb movements, *REM* rapid eye movement, *TST* total sleep time

*Significant

**Fig. 1:**
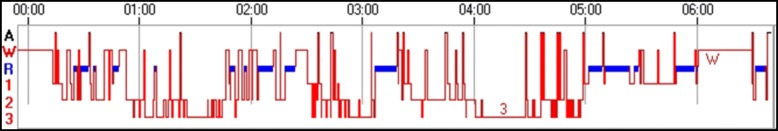
Hypnogram of a diabetic patient with peripheral neuropathy showing sleep fragmentation and increase in each of waking after sleep onset, arousal index, and sleep stage transition index

The mean latencies of MSLT were significantly diminished in DPN group (9.2 ± 4.03 min) compared to DNF and control groups (15.1 ± 3.44 and 16.3 ± 2.2 in respectively) with *p* value < 0.0001. Sleep microstructure showed significant decrease in each of sleep latencies, sleep efficiencies, pulse transit time, lowest O_2_ saturations and REM% of total sleep time (TST) in DPN group compared to DNF and control groups. At the same time, DPN patients showed significant increase in sleep fragmentations, snore % of TST and desaturation, arousal, snore and periodic limb movement indices compared to DNF and control groups (Table 2 and Fig. 1).

**Fig. 2:**
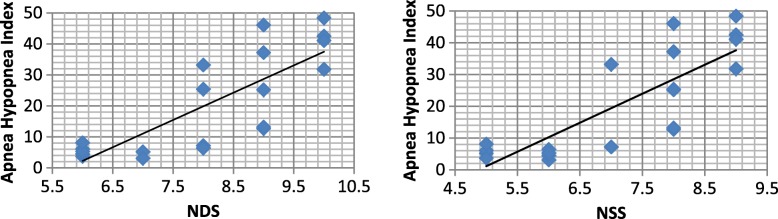
Positive correlation between apnea hypopnea index and each of neuropathy disability score (left) and neuropathy symptom score (right) in diabetic peripheral neuropathy patients

The mean latencies of MSLT in DPN patients showed significant negative correlations with each of diabetes duration, HBA1c, NSS, NDS, postural systolic hypotension, and postural tachycardia. On the other hand, the severity of sleep apnea measured by AHI in DPN patients showed significant positive correlations with each of disease duration, HBA1c, NSS, NDS, postural systolic hypotension, and postural tachycardia (Table 3, Fig. 2).

**Table 3:** Correlations of the multiple sleep latencies and apnea hypopnea indices with other variables in diabetic peripheral neuropathy patients

	MSLT		Apnea hypopnea index	
	r	p	r	*p*
Disease duration	− 0.561	0.0018*	+ 0.703	0.0005*
HBA1c (%)	− 0.663	0.0014*	+ 0.868	< 0.0001*
Neuropathy symptom score	− 0.812	< 0.0001*	+ 0.428	< 0.0001*
Neuropathy disability score	− 0.669	0.0012*	+ 0.806	< 0.0001*
Postural systolic hypotension	− 0.796	< 0.0001*	+ 0.843	< 0.0001*
Postural tachycardia	− 0.474	0.0344	+ 0.843	< 0.0001*

*MSLT* Multiple sleep latency test

*Significant

## Discussion

Sleep apnea syndrome is a disorder in which the airflow suddenly stops or diminishes by > 50% during sleep leading to frequent nocturnal hypoxia and arousals which significantly impairs the sleep quantity and/or quality with consequent deleterious daytime effects including EDS, resistant hypertension, and ischemic heart diseases due to sympathetic overdrive, increased accidents due to decreased attention, cognitive decline, and mood changes (Aurora and Punjabi 2013). Sleep disordered breathing is a common yet under-researched diabetic neuropathy complication with consequent decreased patients’ productivity, increased insulin resistance, and impaired glycemic control (Nannapaneni et al. 2013).

This study showed marked increase in the incidence of sleep disordered breathing in DPN patients (40%) than diabetic patients without neuropathy and healthy control subjects. These results are in accordance with the work of Moon and her colleague (Moon et al. 2015) who found SAS in 50% of their DPN studied patients and attributed this high incidence to impaired bulbar function, reduced pharyngeal tone, diaphragmatic weakness, and even a central component due to impaired chemoreceptors functions. The relatively higher incidence than the present study may be attributed to higher BMI and ages among their studied patients. The last clarification of reduced chemoreceptors functions may be an explanation of the 2 DPN patients who showed mixed obstructive and central sleep apnea in this study.

On the other hand, Meng and his colleagues (Meng et al. 2016) found no relation between the existence of neuropathy in diabetic patients and the presence of sleep complaints. This different result may be explained by their dependence on a subjective sleep quality questionnaire (difficulty falling asleep, early final awakening, short or long sleep) without the use of objective PSG and MSLT. From this difference, we can conclude that many DPN patients may not be aware of their disordered sleep breathing which may only cause frequent micro-arousals not sufficient to awaken the patient. These micro-arousals may not affect the total sleep time but disturb the sleep quality with its diurnal consequences in the form of EDS, cognitive impairment, and mood changes.

The present study had elucidated that most of sleep disordered breathing in DPN was OSA which was evidenced by the high respiratory effort and the significant diminution in pulse transit time during the apnea period. These results agree with that of Nagayoshi and his colleagues (Nagayoshi et al. 2016) and Rasche and colleague (Rasche et al. 2010) who found that OSA is the predominant sleep respiratory disturbance in their studied DPN patients than healthy control subjects, but the incidence of SAS was lower in their works (25% of DPN patients). This difference might be due to the younger ages, shorter disease durations and lower BMI in their included patients which significantly influence the rate of SAS irrespective to the existence of DPN.

The present study declared that, PSG abnormalities are not only higher among DPN patients but also proportional with the severity of sensori-motor manifestations as measured by the NSS and NDS. These data are in accordance with that of Tahrani and his colleagues (Tahrani et al. 2012) and Oleinikov and Sergatskaya (Oleinikov and Sergatskaya 2012). At the same time, they concluded that tight diabetic control cannot be achieved without correction of the OSA by CPAP (continuous positive airway pressure ventilation).

The present study showed that most of DPN patients with sleep disordered breathing had clinical manifestations of dysautonomia either in their complaints (postural dizziness, urinary symptoms, erectile dysfunctions, gustatory sweating, and gastrointestinal symptoms) and/or in examinations (postural systolic hypotension, postural tachycardia, and abnormal heart rate response to deep breath). These results are keeping with each of Dimitropoulos and his colleagues (Dimitropoulos et al. 2014) and Rasche and his colleagues (Rasche et al. 2013) who found a high rate of OSA among DPN patients who had dysautonomia especially that affecting the cardiac parasympathetic and/or sympathetic supply.

At the same time, PSG studying of DPN patients revealed marked impairment of their sleep architecture beyond the sleep apnea in the form of decreased mean latencies of MSLT and sleep efficiencies with markedly increased sleep fragmentations, snore, and periodic limb movements’ indices which may result in impaired sleep quality beyond the diminished sleep time caused by increased walking after sleep onset and repeated arousals. These results were in accordance with that of Cerón and his colleagues (Cerón et al. 2015) and Reutrakul and Mokhlesi (Reutrakul and Mokhlesi 2017) who found a bi-directional relationship between DPN and sleep architecture. The decreased sleep efficiency and the increased sleep fragmentation beside the SAS causes activation of the hypothlamo-pituitary-adrenal axis leading to increased sympathetic overactivity, oxidative stress, and increased systemic inflammation which in turn result in metabolic dysfunction and higher insulin resistance.

## Conclusions

Type 2 diabetic patients complicated by peripheral neuropathy are at increased risk of developing sleep disturbances mainly obstructive sleep apnea, decreased sleep efficiency, sleep fragmentation, and frequent nocturnal hypoxia. These sleep abnormalities significantly disturb patient’s diurnal activities leading to excessive daytime sleepiness, decreased productivity, impaired cognition, mood disturbances, higher rate of accidents, increased insulin resistance, and poor glycemic control. So, early diagnosis and management of sleep disturbances in DPN patients is very important for holistic patient’s management, to increase his productivity, reduce the rate of his diabetic complications, and improve his quality of life.

## Abbreviations

AHI: Apnea hypopnea index
DAN: Diabetic autonomic neuropathy
DM: Diabetes mellitus
DNF: Diabetic neuropathies free
DPN: Diabetic peripheral neuropathy
EDS: Excessive daytime sleepiness
MSA: Mixed sleep apnea
MSLT: Multiple sleep latency test
NDS: Neuropathy disability score
NSS: Neuropathy symptom score
OSA: Obstructive sleep apnea
PSG: Polysomnogram
REM: Rapid eye movement
SAS: Sleep apnea syndromes
TST: Total sleep time
